# Supplementary material for: Preventing pressure injury in nursing homes: developing a care bundle using the Behaviour Change Wheel
Source: BMJ Open. 2019 Jun 3;9(6):e026639. doi: 10.1136/bmjopen-2018-026639 (PMC6561451; doi:10.1136/bmjopen-2018-026639)
Supplement: Supplementary data [file bmjopen-2018-026639supp001.pdf]

**Stage 1: Understand the behaviour**	**Stage 2: Identify intervention options**	**Stage 3: Identify content and implementation options**
1. Define the problem in behavioural terms	5. Identify intervention functions	7. Identify behaviour change techniques
2. Select target behaviour	6. Identify policy categories	8. Identify mode of delivery
3. Specify the target behaviour		
4. Identify what needs to change		

INTERVENTION
