# Supplementary material for: Preventing pressure injury in nursing homes: developing a care bundle using the Behaviour Change Wheel
Source: BMJ Open. 2019 Jun 3;9(6):e026639. doi: 10.1136/bmjopen-2018-026639 (PMC6561451; doi:10.1136/bmjopen-2018-026639)
Supplement: Supplementary data [file bmjopen-2018-026639supp002.pdf]

Pressure Ulcer Prevention Care Bundle

Conduct pressure ulcer risk assessment every month, or sooner if there is a change to resident's health status.
Document findings and due date of next assessment and alert all concerned.

Is the resident:
- At risk?
- At high risk?
- At very high risk?

If **yes**, then…

If **no**, then…

IMPLEMENT CARE BUNDLE

If at **risk**, then **CHECK** and **DOCUMENT ALL** care bundle steps at least every 6 hours

If at **high risk**, then **CHECK** and **DOCUMENT ALL** care bundle steps at least every 4 hours

If at **very high risk**, then **CHECK** and **DOCUMENT ALL** care bundle steps at least every 2 hours

Time – use 24 hour clock 00.00 – 24.00			
**Support surfaces**			
Surface checked for creases, tubing, personal items etc.?			
Equipment checked?			
**Skin inspection**			
All pressure areas checked?			
Redness/changes to skin? Yes (Y) No (N)? (If Y, document overleaf)			
Is the resident experiencing wound pain?			
**Repositioning**			
In bed: rotated onto right (R), left (L) side or hoisted (H)			
Sitting: stood (S) walked (W)			
Other (document overleaf)			
Initials			

Write the frequency here

You will need a new form each day

Initial here

All Care Staff PLEASE READ and ensure all residents 'at risk' have forms in their room. Please ask the nurse in charge to explain if unsure
